# Comparing protective effect of grape seed extract versus atorvastatin on endometriosis in rat model: Evidence for immunohistochemical and biochemical alterations

**Published:** 2015-06-15

**Authors:** Arash Kakaiy, Esmail Ayen, Rajabali Sadrkhanlou, Farshid Sarrafzadeh Rezaei

**Affiliations:** 1 *Department of Theriogenology, Faculty of Veterinary Medicine, Urmia University, Urmia, Iran; *; 2 *Department of Basic Sciences Faculty of Veterinary Medicine, Urmia University, Urmia, Iran; *; 3 *Department of Surgery and Diagnostic Imaging, Faculty of Veterinary Medicine, Urmia University, Urmia, Iran.*

**Keywords:** Atorvastatin, Angiogenesis, Endometriosis, Grape seed extract, Rats

## Abstract

Thirty six Wistar albino rats with implant induced endometriosis were randomly divided into six groups of six animals each. The rats in the first group received nothing and were euthanized at day 21. In the second group, rats received nothing and were euthanized at day 36. The third group received atorvastatin (ATV; 5 mg kg^-1^ per day, orally) until 21 days from induction of endometriosis, and the fourth group received ATV from the 15^th^ day after induction of endometriosis for 21 days. The fifth group received grape seed extract (GET; 450 mg kg^-1^ per day, orally) until 21 days from induction of endometriosis. In the sixth group, GET was administered from the 15^th^ day after induction of endometriosis for 21 days. The estrogen receptor positive cells (ER+) distribution and angiogenesis were assessed using immunohistochemical and immunoflourescent analyzes, respectively. The active cells with intracytoplasmic carbohydrate content were analyzed. Erα mRNA expression was assessed using semiquantitative real time-PCR and the tissue levels of malondialdehyde (MDA), glutathione peroxidase (GSH-px) and superoxide dismutase (SOD) were evaluated. The GET and ATV-treated animals showed significant reduction in endometriosis-increased ER+ cells distribution as well as significant decrease in Erα mRNA levels (*p* < 0.05(. Our data suggests that GET exerts a potent inhibitory effect on development of endometriotic implants similar to ATV.

## Introduction

Endometriosis is the abnormal ectopic growth of cells similar to those that form inside the uterus, consisting of both epithelial and glandular tissues.^1^^,^^2^ According to clinical reports, 6 to 10% of women suffer from endometriosis and pelvic pain, more intensely from infertility problems as well as irregular menstrual cycle.^3^^-^^6^ The most suspected mechanism for endometriosis is that, the endometrial stroma and in some cases epithelial cells migrate outside the uterine horns in retrograde menstruation. According to this theory, the endometrial implants attach ectopically, which in turn result into implants adherence in peritoneal surface.^7^ The attached implants initiate angiogenesis in order to generate neo-vascularization which is necessary for implanted tissue survival.^8^^-^^10^ It has been confirmed that the endometriosis is an estrogen dependent disease and the active estrogen (E_2_) is largely participated in developmental angiogenesis of endometriotic tissue.^11^^,^^12^ Estrogen indirectly activates the vascular growth endothelial factor (VGEF), which stimulates the angiogenesis in implanted tissues. On the other hand, the endometriotic lesions themselves are the source of E_2_, estrone as well as aromatase.^6^^,^^13^ In this regard, it has demonstrated that the aromatase over-expression occurs in endometriotic tissue and its activity is significantly higher than that in normal endometrium.^14^^,^^15^ Therefore, the forced over expression of aromatase, as main enzyme involved in estrogen synthesis, results in considerably higher E_2_ synthesis, which is able to provoke the cellularity in endometrial implants. Thus, we studied Erα+ cells distribution and mRNA level of Erα in grape seed extract (GET) and atorvastatin (ATV)-treated groups in order to compare their inhibitory properties. 

On the other hand, the role of oxidative stress in endometriosis-induced impairment has been reported previously.^16^^,^^17^ Some reports showed that cytokines released from infiltrated macrophages in ectopic endometrium largely influence the redox status. Thus, the cytokines mainly tumor necrosis factor alpha (TNF-α) interact with VGFs in a parallel pathway that results in notable enhancement in tissue levels of glutathione peroxidase (GSH-px) and superoxide dismutase (SOD) as a self-protecting mechanism.^18^^,^^19^ In contrast, some investigators have reported that activated peritoneal macrophages and remarkable polymorphonuclear cells infiltration result in increased oxidative stress in patients.^16^^,^^20^ According to conflicting results, in the present study we tried to clarify the correlation between angiogenesis and the changes in redox system status by comparing the inhibitory effects of GET and ATV on angiogenesis.

Atorvastatin is a member of drug class named statins. The statins are suspected as 3-hydroxy-3-methylglutaryl-coenzyme A (HMG-CoA) reductase inhibitors which lower cholesterol synthesis by blocking conversion of HMG-CoA compounds which exert anti-inflammatory and anti-angiogenesis activities in high dose administration.^22^^,^^23^ Previously, Oktem and co-workers showed that, high dose of ATV regressed the endometriotic implants growth by inhibiting angiogenesis and VGEF expression.^24^

Because of high content of polyphenols, the grape seed has been reported for its anti-cholesterol activities.^25^ Additionally, the grape seed consist of bioflavonoids which are highly antioxidant.^26^ The flavonoid glycosides in grape seed extract are much more rapidly absorbable in human.^27^ Thus, the present study was designed primordially to compare the protective effect of atorvastatin (as a statin compound), on endometriotic implants growth with GET-induced inhibitory effects as anti-cholesterol and antioxidant compound.

## Materials and Methods

**Animals.** Thirty six mature female Wistar rats weighting 250 to 280 gr were used for the experiment. All rats were purchased from Urmia university animal reproduction center and housed in animal laboratory of Urmia University. All animals were acclimated in an environmentally controlled room (20 to 23 ˚C, and 12 hr light/12 hr dark). Food and water were given *ad libitum*. In this study all conducted experiments on animals were in accordance with the guidance of ethical committee for research on laboratory animals of Urmia University.

**Experiment design. **After one week of acclimation the animals were assigned into six groups of six animals each:

**a)** Experimentally endometriosis-induced: Animals euthanized 21 days after endometriosis induction.

**b)** Experimentally endometriosis-induced: Animals euthanized 36 days after endometriosis induction.

**c)** ATV-treated: Animals received ATV (5 mg kg^-1^ per day, orally), until 21 days after induction of endometriosis. 

**d)** ATV-treated: Animals received ATV (5 mg kg^-1^ per day, orally) 15 days after induction of endometriosis for 21 days. 

**e)** GET-treated: Animals received GET (450 mg kg^-1^ per day, orally), until 21 days after induction of endometriosis. 

**f)** GET-treated: Animals received GET (450 mg kg^-1^ per day, orally) 15 days after induction of endometriosis for 21 days. 

**Endometriosis induction.** All the rats were anesthetized with an interaperitoneal administration of 50 mg kg^-1^ ketamine (Rotexmedice, Trittau, Germany) and 7 mg kg^-1^ Xylazine (Alfasan, Woerden, The Netherlands). They were immobilized on a standard rat surgery board. Using the aseptic technique, a ventral midline incision was made to expose the reproductive organs. For inducing endometriosis, the ectopic endometrium was induced surgically as described by Rajkumar *et al*.^28^ Briefly, the left uterine horn was ligated at both the uterotubal junction and the cervical ends using 4-0 silk and removed. A 5 mm segment of the excised horn was cut and placed in sterile isotonic saline. The endometrium was separated from the myometrium and trimmed to 5 × 5 mm. The trimmed section of the endometrium was then transplanted into the peritoneal cavity with the epithelial lining of the segment opposed to the ventro-lateral body wall adjacent to a large vessel using sterile 4-0 silk. The midline abdominal incision was closed with chromic catgut sutures. The skin incision was closed with a horizontal mattress.^28^

**Immunohistochemical staining for estrogen receptor (ER).** The procedure was done according to Jalava *et al*.^29^ Briefly, the commercial mouse monoclonal antibodies were used for ERα (Erα rabbit antimouse, Genova, spain, dilution 1:500). The tissue sections were first deparaffinized with xylene and then washed in descending series of ethanol and TBS-buffer (0.05 M Tris buffered physiological saline, Abcam, USA). Then, the sections were subjected to microwave oven treatment for the retrieval of antigen epitopes (two times for 5 min) in 10 mM aqueous sodium citrate. Then, the antibody was applied. Diaminobenzidine (ScyTek Laboratories, Logan, USA) was used as the chromagen and hematoxylin (Richard-Allen Scientific, Kalamazoo, USA) as the counterstain. Immunohistochemically-stained Erα was analyzed, respectively, using similar principles. The most intensively stained area was chosen using 10 × objective magnification. Then the cells with strong brown positive stained were considered as estrogen receptor alpha positive (ERα+) cells in 40 × objective magnification. Finally the cell number per one mm^2^ of the tissue were obtained and presented.

**Immunoflorescent staining for angiogenesis. **The CD31 immunoﬂuorescence approaches were performed according to established protocol.^30^ In brief, after fixation slides were rinsed three times in 0.1 M phosphate buffered saline (PBS), pH 7.4, for 2 min. Endogenous peroxidase activity was blocked by incubating the slides in 0.3% H_2_O_2_ solution in PBS for 10 min, followed by rinsing slides three times in PBS for 2 min. Fluorescein isothiocyanate (FITC)-conjugated CD31 antibody (Mouse anti rat CD31-FITC, ABD Serotec, MCA1334FB) at 1:80 dilution with PBS was applied directly to the slide and then incubated for 1 hr at room temperature. After rinsing in PBS, biotinylated secondary antibody at 1:50 dilution with PBS was applied directly to the slide for 30 min. Finally, slides were rinsed with water and then dehydrated with alcohol and xylene. In addition, specimens were processed in the absence of the primary antibody as a negative control. The tissue vessels were counted per one mm^2^ of the tissue in 10 slides for each animal and the vessels were characterized in two types of microvasculature (< 5 to 6 µm diameter) and macro-vasculature (> 5 to 6 µm).^31^ Finally, in order to better understand the fluorescent reactions the interactive 3D surface plot for each section obtained by using image pro-insight image analysis software (Version 6.0; Media Cybernetics, Inc., Rockville, USA).

**Reverse transcription polymerase chain reaction (RT-PCR).** Total RNA was removed by precipitation with sodium acetate-isopropanol. The RNA concentration was determined by spectrophotometery and then all samples were DNase treated before reverse transcription. Briefly, 2 μg of total RNA was mixed on ice with 40 U of RNase in, 1 U of DNase and 1X DNase buffer in a final volume of 20 μL. The mixture was left at room temperature for 15 min and the reaction was terminated by adding 2 μL of 25 mM EDTA and heating at 70 ˚C for 10 min. The DNAase-treated sample was divided into two 11 μL aliquots, 100 ng random hexamers were added and the mixture was heated at 70 ˚C for 10 min, immediately chilled on ice for 3 min then centrifuged at 1500 *g* for 1 min. With the tube on ice, the following were added: 1X first strand buffer (25 mM Tris-HCl pH 8.3, containing 37.5 mM KCl and 1.5 mM MgCl_2_), 5 mM DTT and 500 μM dNTP mix (Helix M7501, Promega Co. USA). To one tube (RT + reaction) 200 U superscript II RNase H^- ^reverse transcriptase were added, whereas water was added to the other tube (Control RT-reaction) in a final volume of 20 μL. After gentle mixing, reactions were incubated at room temperature for 10 min then at 42 ˚C for 50 min. Reactions were terminated by heating at 70 ˚C for 15 min.

The PCR reaction was carried out in a programmable thermal cycler (Model 9700; Perkin-Elmer, Warrington, UK). The ERα primer sets used were rERα U (5'-TAAGAACCGG AGGAAGAGTTG) and rERα L (5'-TCATGCGGAATCGACTTG) that give an expected 500 bp product. The relative mRNA expression was normalized using GAPDH (glyceraldehyde-3-phosphate dehydrogenase). The used GAPDH forward and reversed primers were 5’-GGCTGAGAATGGGAAGCT GGTCAT-3’ and 5’-CAGCCTTCTCCATGGTGGTGAAGA-3’, respectively, yielding a 152 bp product. The PCR reactions were then cycled as follows: 5 min at 94 ˚C (1 cycle); 30 sec at 94 ˚C (denaturation step), 30 sec at the 45 ˚C (annealing step) and 1 min at 72 ˚C (extension step) for the required number of cycles (24 cycles for 18 sec and 32 cycles for ERα). Tubes were then incubated for a further 7 min at 72 ˚C (one cycle). Finally, the reaction products were separated on 1.5 % agarose gel and visualized by ethidium bromide staining using Gel Doc 2000 system (Bio-Rad, Hercules, USA). 

**Histomorphometry analyses.** The serial section slides (5 to 6 µm) were prepared and stained using hematoxylin-eosin (H & E) staining. The implanted tissue thickness was measured using morphometric lens devise (Olympus, Hamburg, Germany) in 40 × magnification. The periodic acid–Schiff staining (PAS) was performed in order to analyze the active cells intracytoplasmic carbohydrate ratio. The glandular epithelium and stromal cells reaction for PAS staining was classified into: 0, 1+, 2+ and 3+. The data were presented as mean distribution of cells per 1 mm^2^ of the tissue. In order to reduce the bias problems for staining density, 20 sections for each sample were analyzed.

**Malondialdehyde (MDA)** **determination.** To determine the lipid peroxidation rate, content of the collected implanted tissue samples were measured using the thiobarbituric acid (TBA) reaction as described by Niehaus and Samuelsson.^32^ Samples (0.3 to 0.4 g) were homogenized in 150 mM ice-cold KCL (Sigma Aldrich, St. Louis, USA), and then the mixture was centrifuged at 3,000 *g* for 10 min; 0.5 mL of the supernatant was mixed with 3 mL phosphoric acid (1% v/v) and then following vortex mixing, 2 mL of 6.7 gr per L was added to the samples. The samples were heated at 100 ˚C for 45 min, and then chilled on ice. Finally, 3 mL N-butanol was added and the samples were further centrifuged at 3,000 *g* for 10 min again. The absorbance of supernatant was measured spectrophoto-metrically (S23A; Spectrumlab, Shanghai, China) at 532 nm and the MDA concentration calculated according to simultaneously prepared calibration curves using MDA standards. The amount of MDA was expressed as nmol per mg protein of the samples. The protein content of the samples was measured according to Lowry.^33^

**Assessment of GSH-px and SOD.** After homogenizing the tissues and centrifugation, SOD and GSH-px activities were evaluated using the measurement kits of ransod and ransol kit (Randox laboratories, Crumlin, UK), according to manufactures comment.

**Statistical analyses.** The statistical analyses were performed on all numerical data by using two-way ANOVA and using Origin software (Version 6.0; Microcal Inc., Northampton, USA). All values were expressed as mean ± SD. To compare the graded histological findings between groups, the Kruskal-Wallis test was used. *A p-*value less than 0.05 was considered to be statistically significant. 

## Results

**Fig. 1:**
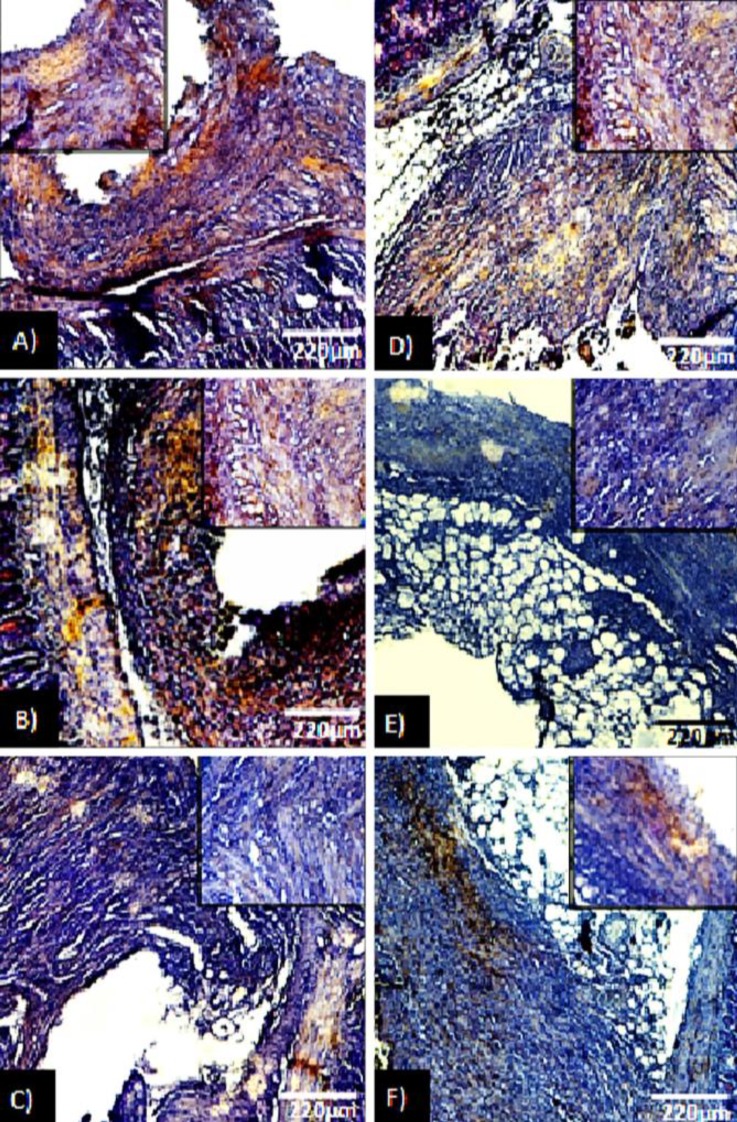
Cross section from ectopic endometrium; **A)** Endometriosis-induced after day 21, **B)** Endometriosis-induced after day 36, **C)** ATV-received until 21 days after induction of endometriosis, **D)** ATV-received from day 15 to day 36 after induction of endometriosis, **E)** Grape seed extract (GET)-treated until 21 days after induction of endometriosis, **F)** GET-treated from day 15 to day 36 after induction of endometriosis. Significant reduction in number of Erα+ cell distribution after 21 and 36 days from endometriosis induction is presented in treated groups (*p *< 0.05), (Immuno-histochemical staining for Erα+ cells, 600× and 800× [inset]).

**Fig. 2:**
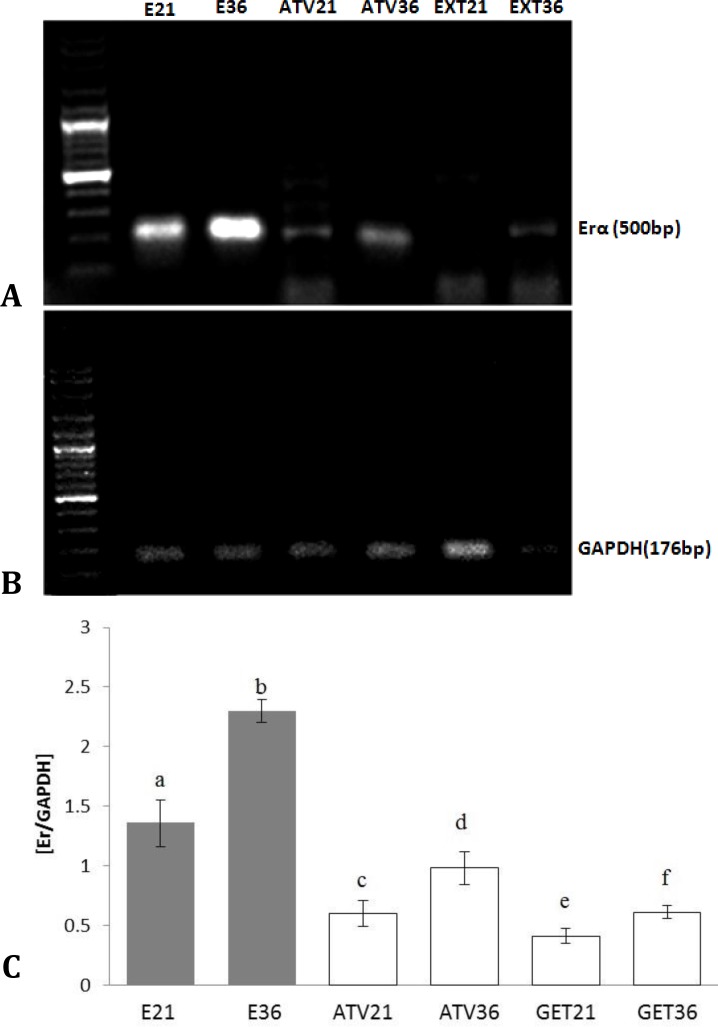
**A)** mRNA levels in the ectopic endometrium tissue, the levels of Erα and GAPDH, **B)** mRNA were evaluated by semi-quantitative RT-PCR, and **C)** Represents the density of Erα mRNA in the endometriotic tissue that were measured by densitometry and normalized to GAPDH mRNA expression level. Results were expressed as integrated density values of Erα mRNA level.

Histological observations demonstrated that, the Erα+ cells distribution was significantly decreased in ATV and GET-administrated groups (*p* < 0.05). Meanwhile, it was more pronounced in GET-treated groups. In contrast, the animals in endometriosis alone groups showed significantly higher number of Erα+ cells per 1 mm^2^ of the tissue, which increased remarkably on day 36 (*p* < 0.05), (Figs. 1A to 1F). Semiquantitative RT-PCR analysis showed the same characteristic. Accordingly, the animals in GET-treated groups were manifested with significant reduction in mRNA level of Erα compared to ATV-treated and non-treated endometriosis-induced groups (*p* < 0.05), (Figs. 2A to 2C).

**Fig. 3:**
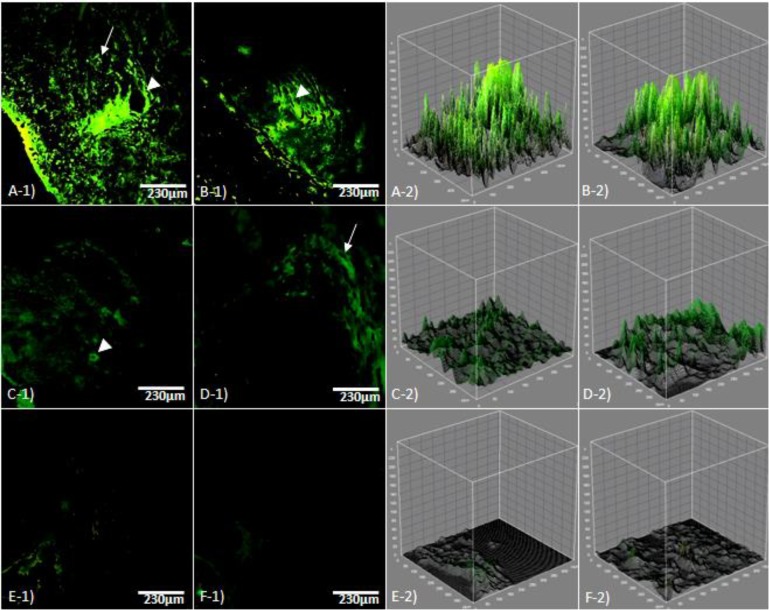
Fluorescent staining for angiogenesis (Arrow: Micro vessels and arrowhead: Macro vessels); **A-1)** Endometriosis-induced after day 21, **B-1)** Endometriosis-induced after day 36, **C-1)** Atorvastatin (ATV)-received until 21 days after induction of endometriosis, **D-1)** ATV-received from day 15 to day 36 after induction of endometriosis, **E-1)** Grape seed extract (GET)-treated until 21 days after induction of endometriosis, **F-1)** GET-treated from day 15 to day 36 after induction of endometriosis. Note significant reduction in vascular distribution in treated groups (*p *< 0.05). The remaining panels indicate scanning plot for light green reactions in 1200 µm, (CD31-FITC conjugated staining, 600×).

**Fig. 4:**
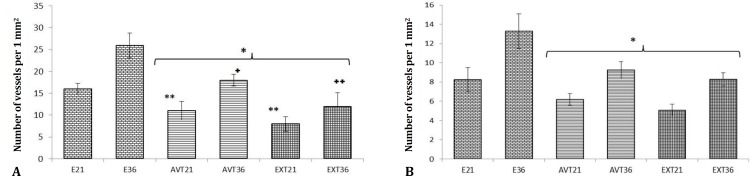
Mean distribution of micro (**A**) and macro-vessels (**B**) in tissue of all groups. All data are presented in mean ± SD and *p *< 0.05 was considered as significant differences. E21: Endometriosis-induced (day 21), E36: Endometriosis-induced (day 36), ATV 21: Atorvastatin (ATV)-treated (day 21), GET21: Grape seed extract (GET)-treated (day 21), ATV36: ATV-treated (day 36) and GET36: GET-treated (day 36).

Immuneflourescent staining for angiogenesis showed that, non-treated endometriosis-induced groups revealed extensional vascular distribution, which was enhanced depending on day. Accordingly, the new generated micro-vesels distribution significantly were increased per 1 mm^2 ^of the tissue (*p* < 0.05). Similarly, the macrovessels expansion was significantly higher in comparison with ATV and GET-treated groups (*p* < 0.05). While the animals which received ATV and GET showed inhibited angiogenesis. Accordingly, the GET-received groups showed the lowest vascular distribution in comparison with AVT-treated groups. Although the micro and macro-vessels distribution lowered in GET-received group, there were no significant differences between ATV and GET-received groups (*p* > 0.05), (Figs. 3A to 3F). The data for micro- and macro- vessels distribution are presented in Figure 4. 

**Fig. 5:**
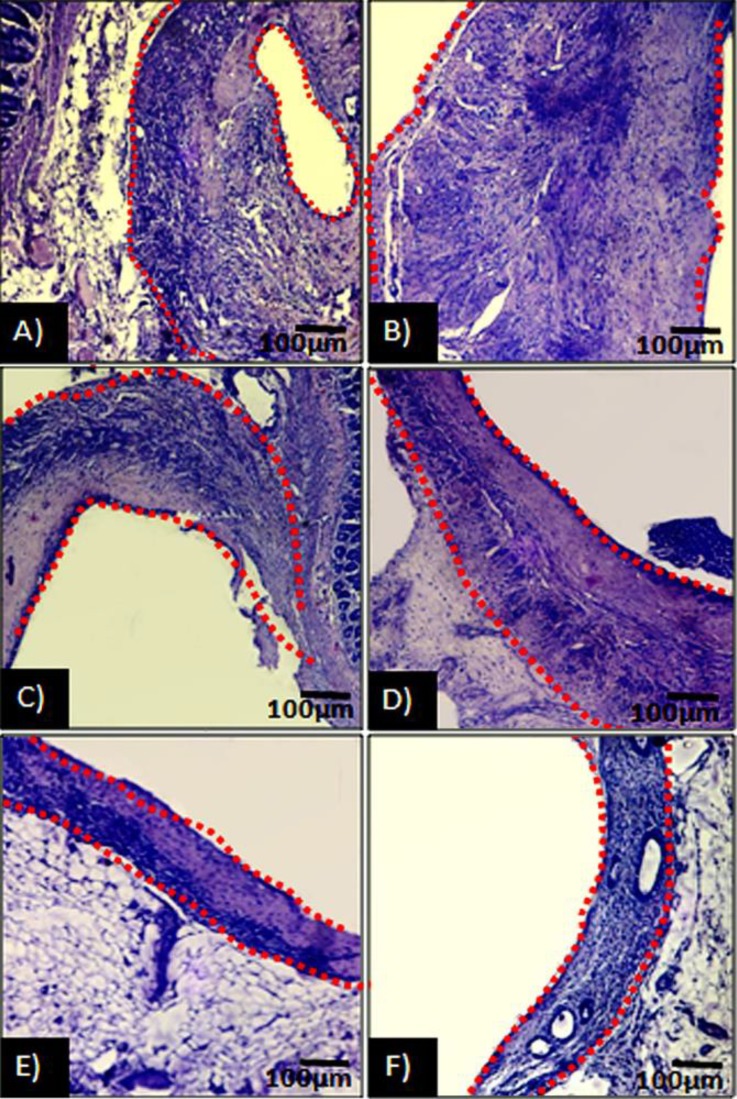
Cross section from ectopic endometrium (Red dots delineated areas); **A)** Endometriosis-induced after day 21, **B)** Endometriosis-induced after day 36, **C)** ATV-received until 21 days after induction of endometriosis, **D)** ATV-received; day 15 to day 36 after induction of endometriosis, **E)** GET-treated until 21 days after induction of endometriosis, **F)** Grape seed extract (GET)-treated from day 15 to day 36 after induction of endometriosis. Note the reduced tissue thickness representing inhibited tissue growth and cellular proliferation in treated groups. GET-treated group showed significant reduction in tissue thickness (*p *< 0.05), (H & E, 600×).

Histomorphometric analyses showed the thickness of the implanted tissue was decreased significantly (*p* < 0.05) in both treated groups. Interestingly, the GET-received animals showed the lowest thickness in comparison with AVT-treated groups on both date of treatment (Figs. 5A to 5F). 

E21: Endometriosis-induced (day 21), E36: Endometriosis-induced (day 36), ATV 21: ATV-treated (day 21), GET21: Grape seed extract (GET)-treated (day 21), ATV36: ATV-treated (day 36), and GET36: GET-treated (day 36).

^ace^ represent significant differences between marked data on day 21 (*p* < 0.05), and ^bdf^ represent significant differences between marked data on day 36 (*p* < 0.05).

* indicates significant difference between treated groups with non-treated groups (*p *< 0.05); while ^+^ and ** show significant differences among marked groups (*p *< 0.05).

**Fig. 6:**
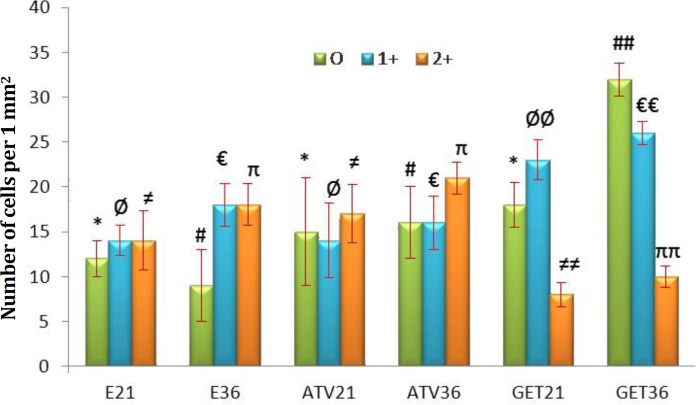
Mean distribution of the 0, 1+ and 2+ PAS-positive cells in 1 mm^2^ of the tissue in different groups. All data are presented in mean ± SD and *p *< 0.05 was considered as significant differences.

**Fig. 7:**
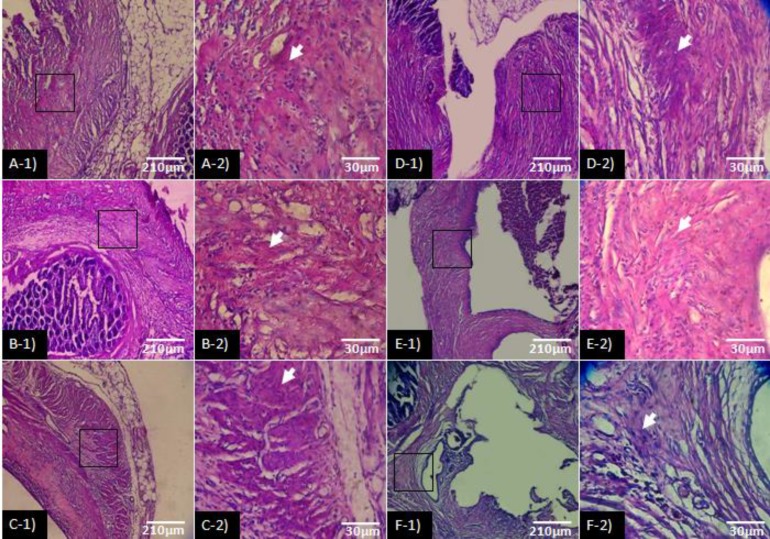
Cross section from ectopic endometrium. Arrows indicate 2+ PAS positive cells. **A)** Endometriosis-induced after day 21, **B)** Endometriosis-induced after day 36, **C)** ATV-received until 21 days after induction of endometriosis, **D)** ATV-received from day 15 to day 36 after induction of endometriosis, **E)** Grape seed extract (GET)-treated until 21 days after induction of endometriosis, **F)** GET-treated from day 15 to day 36 after induction of endometriosis. See higher magnifications in the remaining panels. No significant changes were detectable in non-treated endometriotic and ATV-treated animals, while the cross sections from GET-received groups represented remarkable reduction in 3+ PAS positive cells distribution (*p *> 0.05), (PAS staining, 600× and 800× [inset]).

The special staining for intracytoplasmic carbohydrate content showed that GET remarkably (*p* < 0.05) reduced the 2+ PAS positive cells number per 1 mm^2^ of the tissue (Figs. 6 and 7A to 7F).

**Fig. 8:**
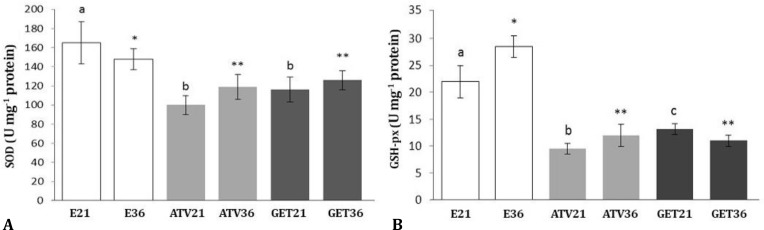
Effect of atorvastatin (ATV) and grape seed extract (GET) on tissue levels (Mean ± SD) of **(A)** glutathione peroxidase (GSH-px) and **(B)** superoxide dismutase (SOD). E21: Endometriosis-induced (day 21), E36: Endometriosis-induced (day 36), ATV 21: ATV-treated (day 21), GET21: GET-treated (day 21), ATV36: ATV-treated (day 36) and GET36: GET-treated (day 36).

^abc^ represent significant differences between marked data on day 21 (*p* < 0.05); * ^and^ ** represent significant differences between marked data on day 36 (*p* < 0.05).

**Fig. 9:**
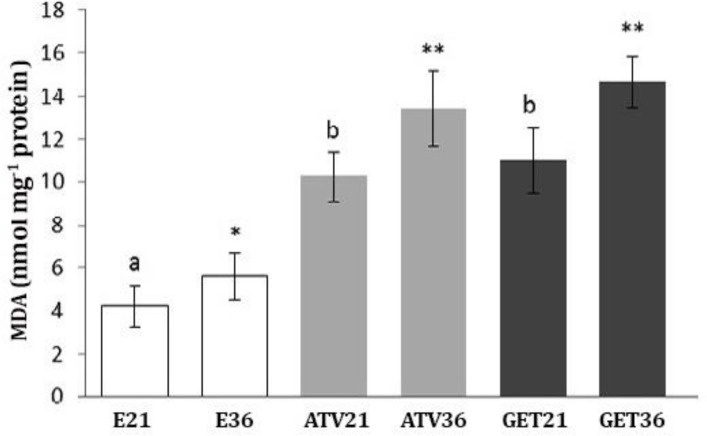
Effect of atorvastatin (ATV) and grape seed extract (GET) on tissue levels (Mean ± SD) of malondialdehyde (MDA).

Biochemical analyses showed that in non-treated endometriosis-induced groups, the GSH-px and SOD levels were significantly higher than those of treated groups (*p* < 0.05). Statistical analyses showed that there were no statistically significant differences between GET and AVT-treated groups after days 21 and 36 (*p* > 0.05), (Figs. 8A and 8B). More assessments on MDA content showed that the tissue level of MDA was significantly lower in non-treated animals compared to treated groups (*p* < 0.05), (Figs. 9). 

E21: Endometriosis-induced (day 21), E36: Endometriosis-induced (day 36), ATV 21: ATV-treated (day 21), GET21: Grape seed extract (GET)-treated (day 21), ATV36: ATV-treated (day 36) and GET36: GET-treated (day 36).

* presenting significant differences between data for E21, ATV21 and GET 21 (*p *> 0.05).

# presenting significant differences between data for E36, ATV36 and GET 36 (*p *< 0.05: # VS ##).

Ø presenting significant differences between data for E21, ATV21 and GET 21 (*p *< 0.05: Ø VS ØØ).

€ presenting significant differences between data for E36, ATV36 and GET 36 (*p *< 0.05: € VS €€).

≠ presenting significant differences between data for E21, ATV21 and GET 21 (*p *< 0.05: ≠ VS ≠≠).

Π presenting significant differences between data for E36, ATV36 and GET 36 (*p *< 0.05: π VS ππ).

E21: Endometriosis-induced (day 21), E36: Endometriosis-induced (day 36), ATV 21: ATV-treated (day 21), GET21: GET-treated (day 21), ATV36: ATV-treated (day 36) and GET36: GET-treated (day 36). ^ab^ represent significant differences between marked data on day 21 (*p* < 0.05); * ^and^ ** represent significant differences between marked data on day 36 (*p* < 0.05)**. **

## Discussion

The present study showed that in non-treated endometriosis condition, the Erα+ cells distribution as well as Erα mRNA level was significantly increased, time dependently. In contrast, administration of ATV and GET resulted in remarkable reduction in proliferation of Erα+ cells. Moreover, more histochemical analyses for intra-cytoplasmic carbohydrates showed that GET was able to reduce the physiologically active cell distribution and could fairly decrease the thickness of implanted tissue. Analyzing the micro- and macro- vessels by immunoflourescent staining clearly showed that both ATV and GET were able to down-regulate the angiogenesis. Finally, mild reduction was detected in GSH-px and SOD levels on day 21 in non-treated endometriosis, while markedly increased angiogenesis ameliorates the anti-oxidant status especially at redox level. However, administration of ATV and GET, albeit with some differences, resulted in considerable reduction in GSH-px and SOD levels and enhanced MDA generation. 

It has been previously shown that the effect of estrogens in endometriotic implants depends on local estrogen biosynthesis, intracellular metabolism of estrogen and finally the microenvironment of endometriotic tissue.^13^ Interestingly, the locally biosynthetized estrogen in ectopic endometriotic tissue exerts higher biological activities with minimal concentration.^13^^,^^34^ In this line, the Erα+ cells play an important role by involving in cellular proliferation under effect of estrogen. The role of Erα in regulating cellular mitogenesis through controlling the cell cycles regulating genes expression (Cyclin D1, C-myc, E2F1, p53 and bcl2) has been reported previously.^35^^,^^36^ Our immunohistochemical and RT-PCR analyses showed that administration of ATV and GET significantly reduced mean distribution of Erα+ cells. At the same time the treated animals exhibited a remarkable reduction in thickness. Thus, we can suggest that GET similar to ATV is able to inhibit the cellular proliferation as well as tissue growth partly by reducing the Erα+ cells population. Via this mechanism, markedly synthesized estrogen’s impression is decreased and in turn leads to prevented tissue growth.

Previous reports showed that estrogen provokes endothelial cell proliferation as well as migration^37^ through classic estrogen receptor (i.e., Erα), which is expressed with endothelial cells.^38^ In this regard, intensive angiogenesis around peritoneal endometriosis implants has been reported in several studies.^8^^,^^9^ Previous reports showed ATV’s anti-angiogenic properties.^9^^,^^39^ Considering decreased angiogenesis in ATV-treated groups, we can come close to this fact that ATV’s exerted impact on Erα+ cells distribution and reduced the tissue growth by inhibiting the angiogenesis. On the other side, it should be noted that GET bears anti-cholestrol and anti-angiogenic properties.^25^ Therefore, diminished angiogenesis after GET administration may be attributed to its effect on cholesterol. Because cholesterol is actively used by endometrial cells as a raw material in the synthesis of estrogen.^40^

Therefore, we can concluded that in addition to GET’s antiangionenic impact, it limited the angiogenesis indirectly via lowering the estrogen synthesis. Ultimately, defected angiogenesis affected the cellular physiologic activities and energy sources. In the light of this fact, the mean number of 2+ and 3+ PAS positive cells were decreased in GET-treated animals. 

Reportedly, the statins directly affect angiogenesis independent to their effect on lipids lowering.^9^^,^^39^ The statins exert two different pro- and anti- angiogenic activities depending on the dose of administration.^39^

In the present study the high dose of ATV was used. Previous findings showed that high doses of ATV induced reductions in geranyl-geranyl pyrophosphate (GGP), which inhibits angiogenesis.^41^ The observations of the present study demonstrated that the ATV-treated animals exhibited reduced angiogenesis which was illustrated with decreased micro- and macro- vessels expansion in endometriotic implants. Thus, we can suggest that ATV administration resulted in endothelial cell apoptosis,^24^ ultimately inhibited angiogenesis. Moreover, ATV inhibited cholesterol synthesis by blocking conversion of HMG-coA to mevalonate and in turn lowered estradiol secretion. The lowered estradiol synthesis associated with endothelial cell apoptosis and resulted in diminished vascularization. This theory was confirmed by significant reduction in serum concentration of estradiol level after ATV administration. The lowered ER+ cells distribution in endometriotic implants confirmed these findings as well. The interesting point was that there were no significant differences between GET and ATV-treated groups for vascular distribution. Gathering together we can come close to this novel theory that controlling estradiol secretion in endometriosis plays an essential role in inhibition of implants angiogenesis, may be much more important than inducing endothelial cell apoptosis by statins.

The role of oxidative stress in etiopathogenesis of endometriosis has been reported. Accordingly, the released cytokines from macrophages in ectopic endometrium enhance the redox status of the cells. ^20^^,^^42^ Indeed, the SOD and GSH-px activity were found to be significantly higher in ectopic endometrium compared with the intact endometrium. In this regard, previous studies showed that the patients with endometriosis exhibited remarkable increase in concentration of TNF-α, which positively correlates with up-regulation of manganese SOD in ectopic endometrium.^18^ Our data showed that administration of ATV and GET significantly decreased SOD and GSH-px content. Considering the degenerative impact of oxidative stress on cellular DNA, RNA, protein and lipid contents, we can conclude that ATV and GET reduced the tissue antioxidant status, which in turn resulted in massive cellular damage. Reduced angiogenesis played synergistic role beside reduced antioxidant power in endometriotic tissue that finally resulted in prevented tissue growth.

In conclusion, the present study showed that GET exerts a potent inhibitory effect on development of endometriotic implants similar to ATV. Moreover, our data showed that GET via its potent anticholesterol and antiangiogenic impacts reduced ERα+ cells distribution, mRNA level as well as neovascularization. Therefore, it could inhibit endometriotic tissue growth similar to ATV. Finally, we showed that GET can be considered as an appropriate novel compound for endometriosis. 
